# Inhibiting Protein Kinase D Promotes Airway Epithelial Barrier Integrity in Mouse Models of Influenza A Virus Infection

**DOI:** 10.3389/fimmu.2020.580401

**Published:** 2020-12-14

**Authors:** Janelle M. Veazey, Sophia I Eliseeva, Sara E. Hillman, Kristie Stiles, Timothy R. Smyth, Charlotte E. Morrissey, Erika J. Tillotson, Dave J. Topham, Timothy J. Chapman, Steve N. Georas

**Affiliations:** ^1^ Department of Microbiology and Immunology, University of Rochester, Rochester, NY, United States; ^2^ Department of Medicine, Pulmonary and Critical Care, University of Rochester, Rochester, NY, United States; ^3^ Department of Environmental Medicine, University of Rochester, Rochester, NY, United States; ^4^ Department Biochemistry, Claremont McKenna College, Claremont, CA, United States; ^5^ Department of Biology, Cornell University, Ithaca, NY, United States; ^6^ Center for Infectious Disease and Immunology, Rochester Regional Health, Rochester, NY, United States

**Keywords:** Airway epithelial barrier, innate immuity, Antiviral immune response, respiratory tract infections, Protein Kinase D

## Abstract

**Rationale:**

Protein kinase D (PKD) is a serine/threonine kinase family that is involved in a wide array of signaling pathways. Although PKD has been implicated in immune responses, relatively little is known about the function of PKD in the lung or during viral infections.

**Objectives:**

We investigated the hypothesis that PKD is involved in multiple aspects of host response to viral infection.

**Methods:**

The selective PKD inhibitor CRT0010166 was administered to C57BL/6 mice prior to and during challenge with either inhaled double-stranded RNA or Influenza A Virus. PKD signaling pathways were investigated in human bronchial epithelial cells treated with CRT0010166, double-stranded RNA, and/or infected with Influenza A Virus.

**Measurements:**

Total protein and albumin accumulation in the bronchoalveolar fluid was used to asses inside/out leak. Clearance of inhaled FITC-dextran out of the airspace was used to assess outside/in leak. Cytokines and neutrophils in bronchoalveolar lavage were assayed with ELISAs and cytospins respectively. Viral RNA level was assessed with RT-PCR and protein level assessed by ELISA.

**Main Results:**

PKD inhibition prevented airway barrier dysfunction and pro-inflammatory cytokine release. Epithelial cells express PKD3, and PKD3 siRNA knock-down inhibited polyI:C induced cytokine production. Lung epithelial-specific deletion of PKD3 (CC10-Cre x PKD3-floxed mice) partially attenuated polyI:C-induced barrier disruption *in vivo*. Mechanistically, we found that PKD promoted cytokine mRNA transcription, not secretion, likely through activating the transcription factor Sp1. Finally, prophylactic CRT treatment of mice promoted barrier integrity during influenza virus infection and reduced viral burden.

**Conclusions:**

Inhibiting PKD promotes barrier integrity, limit pathogenic cytokine levels, and restrict Influenza A Virus infection. Therefore, PKD is an attractive target for novel antiviral therapeutics.

## Introduction

The airway epithelium constitutes a physical barrier to the outside world, and is a key part of host defenses against inhaled viruses (1). Epithelial cells also serve a critical sentinel role, initiating the first wave of cytokines that establish an antiviral state and activate underlying immune cells (2). In addition to inflammation, respiratory viral infections often lead to epithelial barrier dysfunction, but the molecular mechanisms involved remain poorly understood. Understanding how both barrier integrity and early inflammatory signaling are regulated at the molecular level has potential to open the door to new therapeutic targets for a wide array of diseases.

Studies of airway epithelial monolayers *in vitro* have uncovered a role for protein kinase D (PKD) in models of virus-induced barrier dysfunction (3–5). PKD is a serine/threonine kinase family consisting of three isoforms- PKD1, PKD2, and PKD3 (6). PKD has been implicated in a wide range of cellular signaling pathways, including cell growth, differentiation, motility, vesicle secretion, ROS generation, and cytokine production (7–9). The PKD3 isoform was recently identified as mediating airway epithelial barrier disruption in 16HBE cells, a human bronchial epithelial cell line (4). However, the expression or function of PKD3 in the lung has not been previously reported.

Emerging evidence points to a role for PKD in immune responses to infectious pathogens. For instance, PKD1 was shown to promote pro-inflammatory cytokine release in cell lines exposed to flagellin (10), and also to promote cytokine production and leukocyte accumulation into the mouse lung following bacterial challenge (11, 12). In myeloid cells, PKD was shown to be activated downstream of the adaptor MyD88 (11, 13), which is activated by an array of non-viral pathogens. In contrast, very little is known about the function of PKD in viral infections. Here we investigated the expression and function of PKD in airway epithelial cells in models of respiratory viral infection. We report a novel role for airway epithelial PKD3 in regulating pulmonary barrier integrity *in vivo* and identify PKD as a potential therapeutic target during viral infection.

## Methods

### Cell Lines, Mice, Virus

#### Cell Lines

16HBE14o- human bronchial epithelial cells (a gift from Dr. D. C. Gruenert, University of California San Francisco, CA) were cultured in minimum essential medium supplemented with 10 mmol/L HEPES, 10% FBS, and glutamine.

#### Mice

C57BL/6NCr mice were obtained from the National Cancer Institute. All animals were treated according to the Institutional Animal Care and Use Committee at the University of Rochester. PKD3 floxed mice were generated by homologous recombination using targeting vectors with lox P sites flanking exon 3 at the Mouse Genomics Core services at the University of Rochester, NY. Genotyping strategies are indicated in **Supplementary Figure 3**. CC10-Cre mice (14) were a generous gift from Dr. Tom Mariani at the University of Rochester, NY. MDA5^-/-^ mice on the C57BL/6 background (**Supplemental Figure 2**) were purchased from Jackson Labs and bred for experiments at the University of Rochester. All animals were treated according to the Institutional Animal Care and Use Committee at the University of Rochester.

#### Virus

Influenza A Virus (IAV) strain A/PR/8/1934(H1N1) (hereafter referred to as PR/8) was a generous gift from Dr. Dave Topham at the University of Rochester, NY.

### Reagents and Kits

All assays were used according to manufacturer’s instructions.

**Table:** 

Reagent	Company	Catalogue Number
High molecular weight polyI:C	InvivoGen	Version#11C21-MM
PKD inhibitor CRT0066101 (CRT)	Tocris	4975
PKD inhibitor CID755673 (CID)	Tocris	3327
Murine CXCL1 ELISA	R&D Systems	DY453
Murine IFN-lambda ELISA	R&D Systems	DY1789
Murine Albumin ELISA	AbCam	Ab108792
Th1/Th2 cytokine 8-plex mouse ProcartaPlex 2	Invitrogen	#EPX080-20832-901
Human IL-8 ELISA	R&D Systems	DY208
Human IFN-lambda ELISA	R&D Systems	DY1598B
Influenza A virus HA protein ELISA	Sino Biological	SEK11684
4kDa FITC-dextran	Sigma	46944
Golgi Plug	BD Bioscience	555029
Transcription Factor Activation Profiling Plate Array 1 Kit	Signosis	FA-1001
Sp1 binding assay	RayBiotech	P08047

### Assay of Inside-Out Leak

Inside-out leak refers to the transudation of macromolecules from the circulation or interstitial space into the airway lumen. To assess inside-out leak, C57BL/6 mice were treated with the PKD inhibitor CRT two days prior to receiving 10 ug polyI:C, days 0–2. Both CRT and polyI:C were administered by oropharyngeal aspiration (o.p.). Mice were euthanized 24 h after final polyI:C challenge and bronchoalveolar lavage (BAL) fluid was analyzed for total protein (Bradford), albumin (ELISA), cytokines (ELISAs and Multiplex), and neutrophils (cytospin).

### Assay of Outside-In Leak

Outside-in leak refers to the passive transudation of macromolecules out of the airway lumen. To assess outside-in leak, C57BL/6 mice were administered CRT and polyI:C as above. On day 3, 0.2mg 4kDa FITC-dextran was administered o.p 1 h prior to harvest, as previously reported (15). FITC-dextran levels in BAL fluid and serum were analyzed. with a plate reader (Beckman Coulter DTX 880 Multimode).

### siRNA Constructs

For transfections, 16HBE cells at 60% confluency were treated with 20 pmol siRNA directed against PKD3 or scramble control and Lipofectamine 3000 in serum free media (OptiMem). After 18 h, transfection media was removed and replaced with complete DMEM+10% FBS for 24 h to recover and reach 100% confluency before exposure to polyI:C and/or CRT. Media was collected for cytokine analysis (ELISA) and cells lysed for either protein (Western blot) or RNA (RT-PCR) analysis. The following constructs were purchased: Control-siRNA (Qiagen no. 1022076), PKD3 siRNA (Qiagen no. SI02223984), Sp1 siRNA (ThermoFischer, no 116546).

### Western Blot Antibodies

All primary antibodies were probed overnight at 4°C in 5% bovine serum albumin (BSA). Secondary antibodies were probed 2 h at room temperature in 5% milk. Bands were visualized with Clarity Enhanced Chemiluminescence (BioRad).

**Table:** 

Antibody	Company	Dilution
Anti-PKD1	Santa Cruz A-20; sc-638	1:500
Anti-PKD2	Cell Signal Technology 8188	1:1000
Anti-PKD1/2	Cell Signal Technology 90039	1:1000
Anti-PKD3	Cell Signal Technology 5655	1:1000
Anti-pMOTIF	Cell Signal Technology 4381	1:750
Anti-phospho-p56/NF-kB	Cell Signal Technology 3033	1:500
Anti-p65/NF-kB	Invitrogen 43670	1:1000
Anti-phospho-p38/MAPK	Cell Signal Technology 9211	1:500
Anti-p38/MAPK	Invitrogen 33-1300	1:1000
Anti-GAPDH	AbCam 8245	1:50,000

### RT-PCR

Cells were lysed according to Omega R.N.A.E.Z. kit (R6834-02). 0.5 ug RNA was used to make cDNA and 1/10th of cDNA was used per RT-PCR reaction. Primer sequences are listed in supplemental. The cycle protocol was 94°C 2min; 94°C 30s, 48°C 60s, 72°C 45s x40cycles.

**Table:** 

Primer	Number of Bases​	Sequence
hIL-29(IFN-lambda1)-F’	20	GTTCAAATCTCTGTCACCAC
hIL-29(IFN-lambda1)-R’	20	TTCAGCTTGAGTGACTCTTC
hIL-8 Forward	20	CTTGGCAGCCTTCCTGATTT
hIL-8 Reverse	20	CAGCCCTCTTCAAAAACTTC
hPKD1 For	20	TGCCAGAGCACATAACGAAG
hPKD1 Rev	20	TTCTCCCACCTCAGGTCATC
hPKD2 For	20	CAACCCACACTGCTTTGAGA
hPKD2 Rev	20	CACACAGCTTCACCTGAGGA
hPKD3 For	18	CGGAGCAAAGGTTACAAC
hPKD3 Rev	18	AAGCCAAGTCTGATAGTCCTG
hGAPDH-Forward	19	ACATCGCTCAGACACCATG
hGAPDH-Reverse	22	TGTAGTTGAGGTCAATGAAGGG

### Statistical Analysis

All values are expressed as means ± standard deviation. Statistical analyses were performed using an unpaired t-test for two groups and ANOVA followed by Tukey’s multiple comparisons test. A p-value of 0.05 or less was considered statistically significant. All data were analyzed using GraphPad Prism.

## Results

### Inhibiting PKD Limits Both Inside/Out and Outside/in Leak *In Vivo*

PKD was previously implicated in polyI:C-induced barrier dysfunction in human bronchial epithelial cell monolayers *in vitro* (3, 4). PolyI:C is a synthetic dsRNA, and activates different nucleic acid sensing pathways in epithelial cells. In order to determine if PKD was involved in dsRNA-induced barrier dysfunction *in vivo*, we used the water-soluble competitive PKD inhibitor CRT0066101 (referred to as CRT from here on) (16). CRT has been found to be specific for PKD in an assay of over 90 kinases, and has an IC_50_ for PKD1, PKD2, and PKD3 of 1, 2.5 and 2nM *in vitro*, respectively (16). We found that concentrations of CRT up to 5 uM did not exhibit significant *in vitro* toxicity as measured by LDH assay (data not shown).

**Figure 1:**
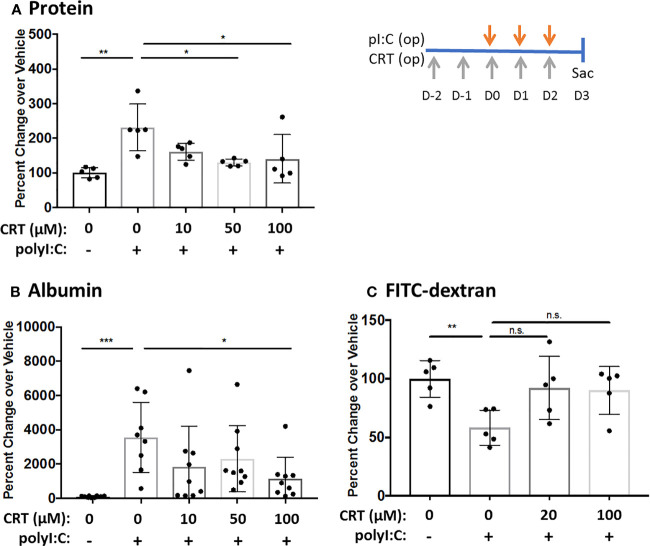
Inhibiting protein kinase D (PKD) limits both inside-out and outside-in leak *in vivo*. C57BL/6 mice were administered vehicle or 10–100 μg CRT by oropharyngeal inhalation (o.p.) on D-2 and D-1. On D0-2 mice were administered CRT plus 10 μg polyI:C. BAL fluid was harvested on D3 and analyzed for total protein level *via* Bradford assay **(A)** and albumin *via* ELISA assay **(B)**. For FITC-dextran flux: 1hr prior to harvest mice were administered 0.2mg 4kDa FITC-dextran o.p. and BAL fluid was analyzed with fluorescence plate reader **(C)**. Data are mean ± standard deviation. Each point represents an individual mouse from 2–3 independent experiments. One way ANOVA followed by unpaired Tukey’s multiple comparisons test. *p < 0.05, **p < 0.01, ***p < 0001. ns=p>0.05.

We first validated that polyI:C dose-dependently increased intracellular PKD activity as determined by Western blot analysis of phosphorylated PKD substrates (17), and that this was blocked by CRT (**Supplemental Figure 1**). We then asked if CRT treatment could limit airway barrier disruption caused by dsRNA *in vivo* using a polyI:C inhalation challenge protocol (15). **Figure 1A** shows that CRT significantly and dose-dependently reduced the amount of total protein and albumin translocation into the airspace after polyI:C challenge, which are markers of inside/out leak. Similarly, **Figure 1B** shows that CRT treatment significantly prevented the rapid loss of 4kDa FITC-dextran out of the airspaces, which is a direct reflection of epithelial barrier function and a marker of outside/in leak (15). CRT did not inhibit the basal levels of BAL albumin detected in PBS challenged control mice (104 ± 52 vs. 131 ± 87 pg/ml, n=4, PBS vs. PBS plus CRT, p>0.05). These data indicate that inhaled polyI:C promotes bidirectional leak in the lung, and inhibiting PKD limits the degree of leak in both directions.

### Inhibiting PKD Limits Neutrophil Influx and Pro-Inflammatory Cytokine Levels *In Vivo*

**Figure 2:**
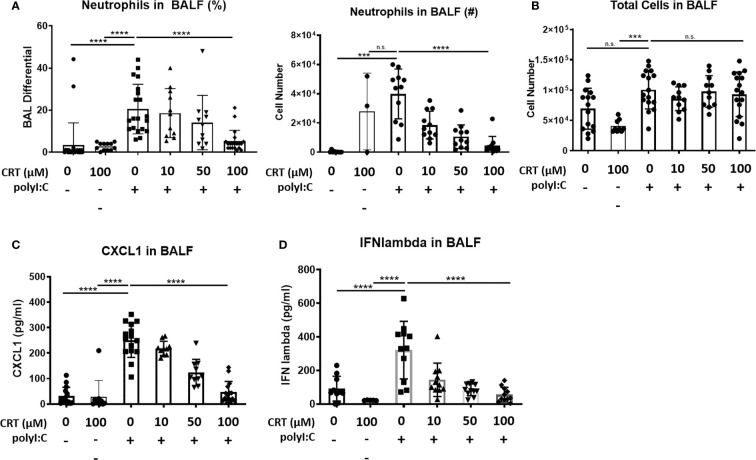
Inhibiting protein kinase D (PKD) limits CXCL1 levels and attenuates neutrophil influx into the lung following polyI:C inhalation. C57BL/6 mice were administered vehicle or 10–100 μg CRT o.p. days -2, -1. On days 0–2, mice were administered CRT plus 10 μg polyI:C. BAL fluid was harvested on day 3 and analyzed for **(A, B)** neutrophil percentages, number, and total cell number, using cytospin, and **(C)** CXCL1 and **(D)** IFN-lambda levels by ELISA. Data are mean ± standard deviation. Each point represents an individual mouse from 3 independent experiments. One way ANOVA followed by unpaired Tukey’s multiple comparisons test. ***p <0.001, ****p<0.0001. ns=p>0.05.

Interestingly, we also found that inhibiting PKD reduced the influx of inflammatory cells into the airspaces after polyI:C inhalation. **Figure 2** shows that three days of inhaled polyI:C promoted robust neutrophil accumulation into the airspace (7.8 ± 16.1% vs. 25.6 ± 14.2%, in saline vs. polyI:C treated mice as determined by cytospin of BAL fluids). Neutrophils are the only inflammatory cell time detected by cytospin at these time points, and CRT treatment dose-dependently attenuated polyI:C-induced neutrophil accumulation, but not total leukocyte numbers (**Figures 2A, B** **)**. CRT treatment also dose-dependently attenuated polyI:C-induced levels of the neutrophil attracting chemokine CXCL1 in BAL fluid (**Figure 2C**), as well as levels of the antiviral cytokine IFN-lambda (**Figure 2D**). CRT did not inhibit the basal levels of CXCL1 detected in response to PBS challenge in control mice (4 ± 3 vs. 7 ± 6 pg/ml, n=5, PBS vs. PBS plus CRT, p>0.05).

**Figure 3:**
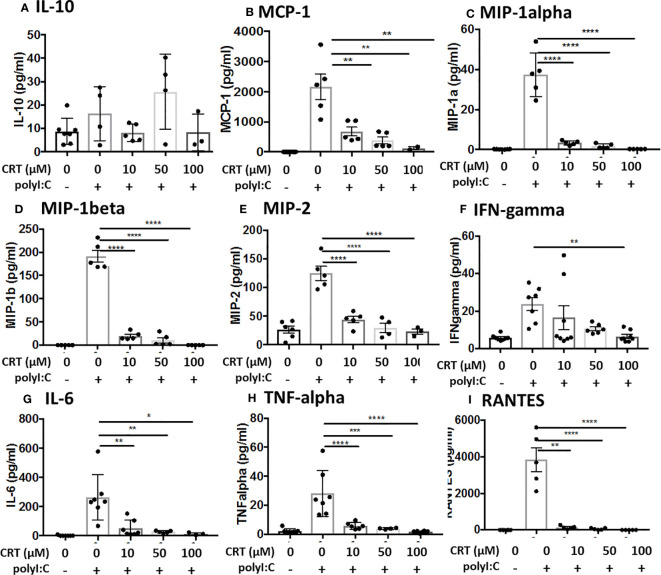
Inhibiting protein kinase D (PKD) limits several pro-inflammatory cytokines. C57BL/6 mice were administered vehicle, CRT or CRT plus polyI:C as in the legend to **Figure 2**. BAL fluid was harvested D3 and analyzed for chemokines/cytokines using multiplex bead array inlcuding **(A)** IL-10, **(B)** MCP-1, **(C)** MIP-1alpha, **(D)** MIP1-beta, **(E)** MIP-2, **(F)** IFN-gamma, **(G)** IL-6, **(H)** TNF-alpha, and **(I)** RANTES. Data are mean ± standard deviation. Each point represents an individual mouse from 2 independent experiments. One way ANOVA followed by unpaired Tukey’s multiple comparisons test. *p < 0.05, **p < 0.01, ***p < 0.001, ****p < 0.0001.

These results suggested that PKD inhibition might have a broader effect on polyI:C-induced cytokine and chemokine production. To investigate this possibility, we analyzed BAL fluid using a multiplex array and found that PKD inhibition significantly reduced the levels of several pro-inflammatory cytokines including IL-6, MIP1-alpha, MIP1-beta, and MIP2 in a dose-dependent manner (**Figure 3**). Levels of IL-10 were not significantly inhibited by CRT (**Figure 3**). Taken together, these data indicate that in addition to promoting epithelial barrier disruption in response to inhaled dsRNA, PKD also enhances pro-inflammatory cytokine and chemokine production, as well as neutrophil recruitment to the lung.

As CRT treatment limits both barrier disruption and neutrophil influx, it was possible that CRT promoted barrier integrity indirectly by limiting airway inflammation, or directly by acting on airway epithelial cells. To try and distinguish between these possibilities (which are not mutually exclusive), we revisited our previously published model using wild-type, and TLR3- and MDA5-gene-targeted mice (15). In this model, we found that barrier integrity is mediated by TLR3, while acute neutrophil accumulation (but not barrier disruption) was attenuated in MDA5 knock-out mice. We reasoned that if CRT promoted barrier indirectly by limiting neutrophil levels, then CRT treatment of MDA5^-/-^ mice (where neutrophil levels are reduced) would not further promote barrier integrity. However, we found that CRT treatment restored barrier integrity equally well in both wild-type and MDA5-gene targeted mice after polyI:C challenge (**Supplemental Figure 2**). Therefore, these results support the idea that inhibiting PKD signaling restores barrier function independently of reducing airway neutrophil influx.

### PKD3 Is Highly Expressed in the Lung and in 16HBE Cells, and Promotes PolyI:C-Induced Cytokine Secretion

**Figure 4:**
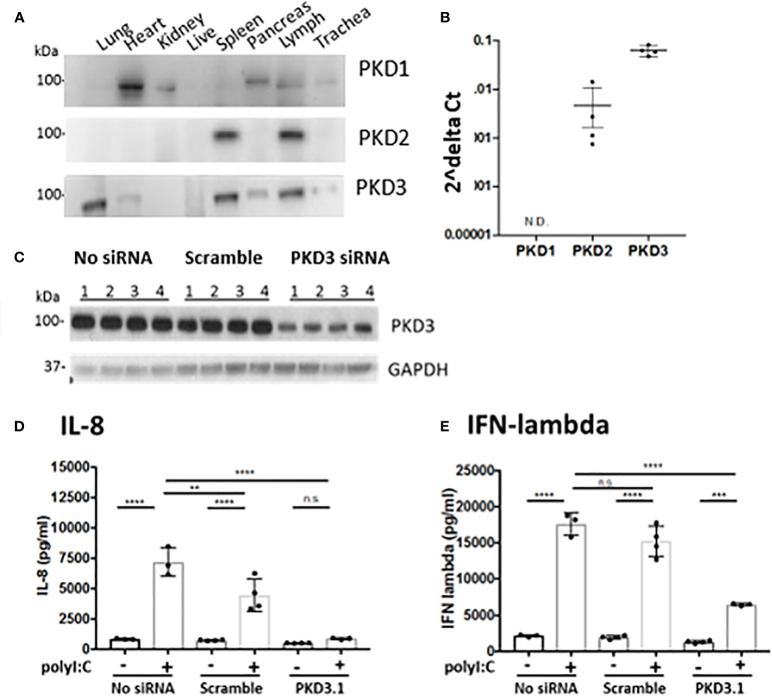
PKD3 is highly expressed in the lung and in 16HBE cells and promotes cytokine secretion. **(A)** Tissues from untreated C57BL/6 mice were analyzed for PKD isoforms *via* Western blot. **(B)** RNA was harvested from confluent16HBE monolayers and levels of the PKD isoforms were quantified with RT-PCR and normalized to GAPDH. **(C)** 16HBE cells were transfected with 20 pmol siRNA targeting PKD3 prior to 6hr stimulation with 5 μg/ml polyI:C. siRNA-mediated knock down of PKD3 in 16HBE cells was confirmed *via* Western blot, and supernatants were analyzed for IL-8 **(D)** and IFN-lambda **(E)** *via* ELISA. Data are mean ± standard deviation. Each point represents an individually treated well from one experiment. One way ANOVA followed by unpaired Tukey’s multiple comparisons test. **p < 0.01, ***p < 0.001, ****p < 0.0001. ns=p>0.05.

Western blot analysis of PKD expression in different mouse tissues confirmed that different PKD isoform are expressed in a tissue-specific manner (**Figure 4**). We found elevated expression of PKD1 in the heart and pancreas, and PKD2 in immune organs, consistent with prior studies (9). By contrast, PKD3 was highly expressed in the lung, spleen and lymph nodes compared with other tissues. Furthermore, neither PKD1 nor PKD2 was readily detected in the lung (**Figure 4A**). In 16HBE cells, we similarly found that PKD3 mRNA transcripts were ~100 fold more abundant than PKD2 transcripts, and PKD1 transcripts were below the detection limit of the assay (**Figure 4B**).

Since we found PKD3 to be the predominant isoform in both the lung and airway epithelial cells, we next investigated the role of PKD3 in dsRNA-induced cytokine production in 16HBE cells, since these cells can be readily transfected with siRNA constructs. Consistent with this hypothesis, siRNA-mediated knock down of PKD3 (confirmed by Western blot analysis, **Figure 4C**) attenuated polyI:C-induced secretion of both IL-8 and IFN-lambda (**Figures 4D, E** **)**. PKD3 siRNA knock-down did not significantly inhibit the low levels of basal cytokine production detected from unstimulated cells (p>0.05). Taken together, these data implicate a novel role for PKD3 in promoting cytokine/chemokine production in airway epithelial cells exposed to dsRNA.

### PKD3 in Airway Epithelial Cells Is Sufficient to Regulate Barrier Disruption *In Vivo*

**Figure 5:**
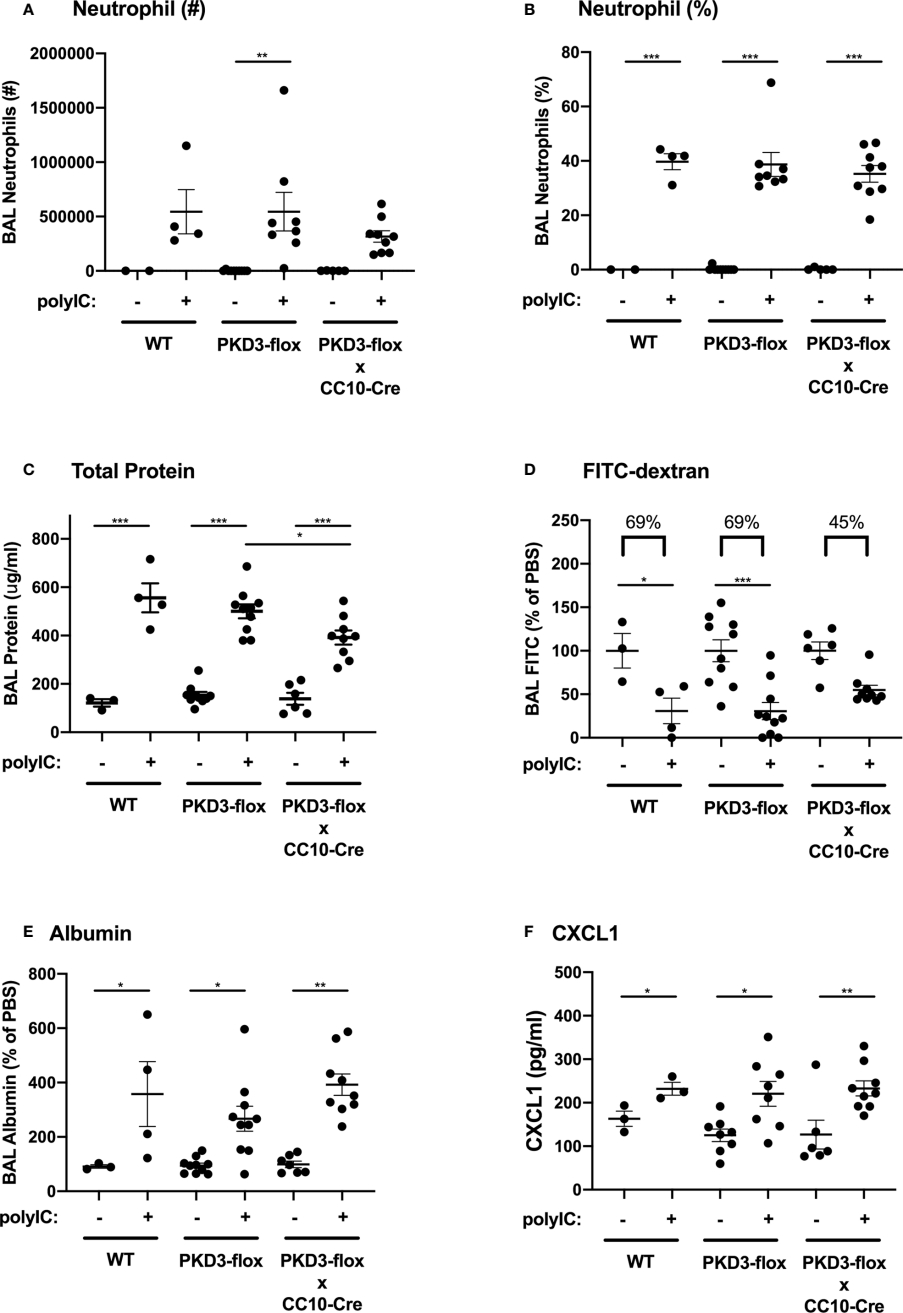
PKD3 in airway epithelial cells contributes to polyI:C-induced barrier disruption *in vivo.* Wild-type (WT), PKD3-flox, or dual transgenic PKD3-flox X CC10-Cre mice were administered vehicle or 10 μg polyI:C days 0–2. BAL fluid was harvested day 3 and analyzed for neutrophils numbers and percentages **(A, B)**. Inside/out barrier function was determined by measuring total protein and albumin levels in BALF **(C, E)**, and outside/in barrier function was measured by analyzing the rapid clearance of FITC-dextran out of the airspaces **(D)**. CXCL1 levels were also measured **(F)**. Data are mean ± standard deviation. Each point represents an individual mouse from 2 independent experiments. One way ANOVA followed by unpaired Tukey’s multiple comparisons test. *p < 0.05, ***p < 0.001, ****p < 0.0001.

We next investigated whether PKD3 expression in the airway epithelium contributed to the observed dsRNA-induced responses in our mouse model. To test this, we generated PKD3-floxed mice and crossed them with CC10-Cre mice to establish a conditional deletion of PKD3 in CC10+ airway epithelial Club cells. After confirming successful deletion of PKD3 in the lung (**Supplemental Figure 3**), we challenged PKD3 conditional knock out mice with polyI:C as described in **Figure 1**. Wild-type and single transgenic PKD3-floxed mice were used as controls. PolyI:C-induced neutrophil influx was not significantly different between groups (**Figures 5A, B**). Total protein leak into the BAL fluid was lower in CC10-Cre x PKD3-floxed mice compared to single transgenic PKD3-floxed controls (**Figure 5C**), although BAL albumin levels were similar between groups (**Figure 5E**). While both wild type mice and single transgenic PKD3-floxed control mice demonstrated significant FITC-dextran leak out of the airspace following polyI:C treatment (~69% reduction, **Figure 5D**), FITC-dextran loss was attenuated in CC10-Cre x PKD3-floxed mice (~45% reduction, **Figure 5D**). Finally, polyI:C-induced CXCL1 production was not affected by epithelial-specific PKD3-deletion (**Figure 5F**). Together, these data indicate that PKD3 promotes polyI:C-induced barrier dysfunction in the lung at least in part in an epithelial cell intrinsic manner.

### PKD Activates Sp1 to Promote Cytokine Production at the Level of mRNA Transcription

**Figure 6:**
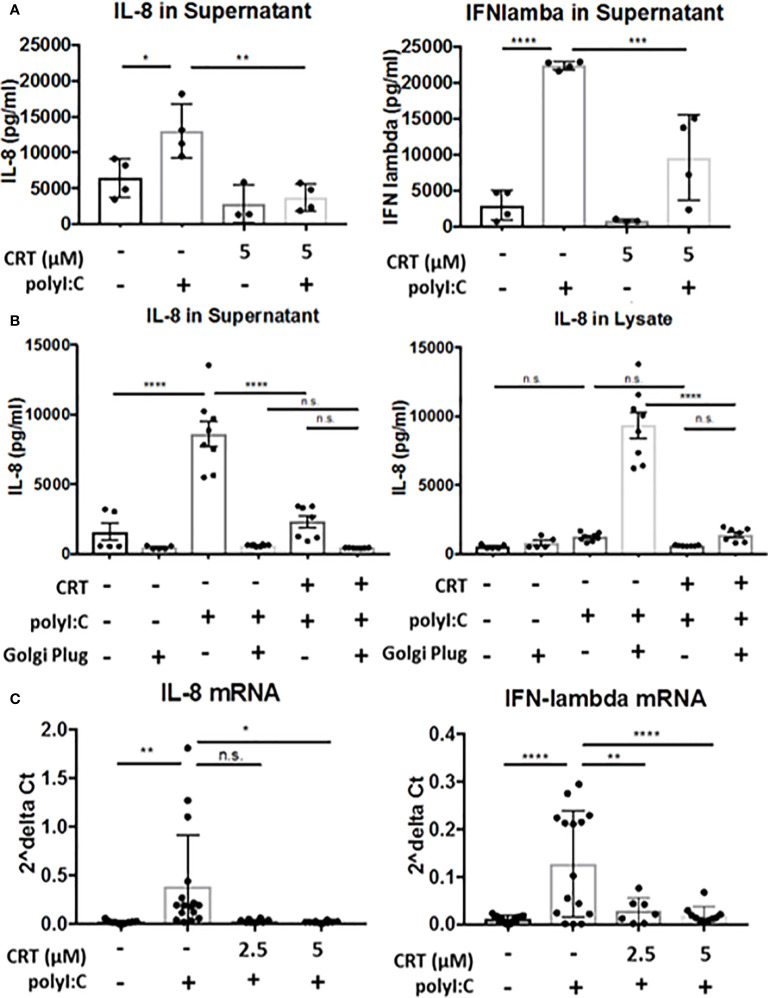
Protein kinase D regulates cytokine production, not secretion. **(A)** 16HBE cells were pretreated for 2 h with 5 μM CRT before polyI:C was added to a final concentration 5 μg/ml. After 6 h, supernatant was analyzed for IL-8 or IFN-lambda *via* ELISA (R&D Bioscience). **(B)** 16HBE cells were treated with Golgi Plug for 30 min. prior to adding in CRT to a final concentration of 5 μM. 2 h later polyI:C was added to a final concentration of 5 μg/ml. After 6 h, supernatant and whole cell lysate was analyzed for IL-8 *via* ELISA (R&D Bioscience). **(C)** 16HBE cells were pretreated 2h with 2.5-5 μM CRT before polyI:C was added to a final concentration 5 μg/ml. RNA was isolated using E.Z.N.A. kit (Omega). RT-PCR was performed from 0.5 μg cDNA per sample. Data are mean ± standard deviation. Each point represents an individually treated well from 1–3 independent experiments. One way ANOVA followed by Tukey’s t-test. *p < 0.05, **p < 0.01, ***p < 0.001, ****p < 0.0001. ns=p>0.05.

We next explored the molecular mechanisms by which PKD regulates cytokine production in airway epithelial cells. We used monolayers of human 16HBE epithelial cells for these experiments, since these cells form a polarized barrier *in vitro* and can be readily transfected with siRNA constructs. Similar to results from polyI:C challenged mice, polyI:C treatment of 16HBE cells induced robust levels of the neutrophil chemoattractant IL-8, the functional homologue to mouse CXCL1 (6398 ± 2,687pg/ml vs. 13,001 ± 3,759pg/ml, vehicle vs. polyI:C), which was markedly attenuated by CRT (2,824 ± 2,593pg/ml, p<0.05). Production of IFN-lambda was similarly induced by polyI:C (2,993 ± 2,063pg/ml vs. 22,377 ± 585pg/ml, vehicle vs. polyI:C), and inhibited by CRT (871 ± 191pg/ml, p<0.05) (**Figure 6A**). These results were confirmed with a second structurally unrelated PKD inhibitor, CID (**Supplemental Figure 4**).

PKD has been previously implicated in regulating both vesicular traffic at the Golgi apparatus, as well as in transcriptional regulation of gene expression (9, 18). We next asked if inhibiting PKD with CRT lowers cytokine secretion or production. To assess cytokine secretion, we treated cells with or without CRT and Golgi Plug, a potent inhibitor of protein transport from the Golgi complex. We reasoned that if PKD regulated Golgi-dependent cytokine secretion in airway epithelial cells, then levels of intracellular and extracellular cytokine from CRT-treated cells would be similar to that of cells treated with Golgi Plug. Instead, we found that CRT-treated cells exhibited less IL-8 intracellularly compared to Golgi Plug treated cells (5,926 ± 1,355pg/ml vs. 333,158 ± 6,601pg/ml in CRT+polyI:C-treated vs. Golgi Plug+polyI:C-treated cells). CRT treatment also significantly lowered polyI:C-induced cytokine released into the supernatant compared to vehicle-treated cells (14,388 ± 2,406pg/ml vs. 32,868 ± 7,049pg/ml in CRT+polyI:C treated vs. vehicle+polyI:C treated cells, p<0.05) (**Figure 6B**). Similar results were obtained with IFN-lambda (**Supplemental Figure 5**). In unstimulated 16HBE cells, CRT treatment did not significantly inhibit constitutive production of either cytokine (p>0.05). These data suggest that PKD is involved in cytokine gene expression rather than Golgi-dependent secretion. We next asked if PKD regulated cytokine production at the level of mRNA transcription. We found that CRT-treatment essentially abrogated the induction of IL-8 and IFN-lambda mRNA following polyI:C stimulation (**Figure 6C**). Together these data demonstrate that PKD promotes cytokine mRNA transcription rather than protein secretion.

**Figure 7:**
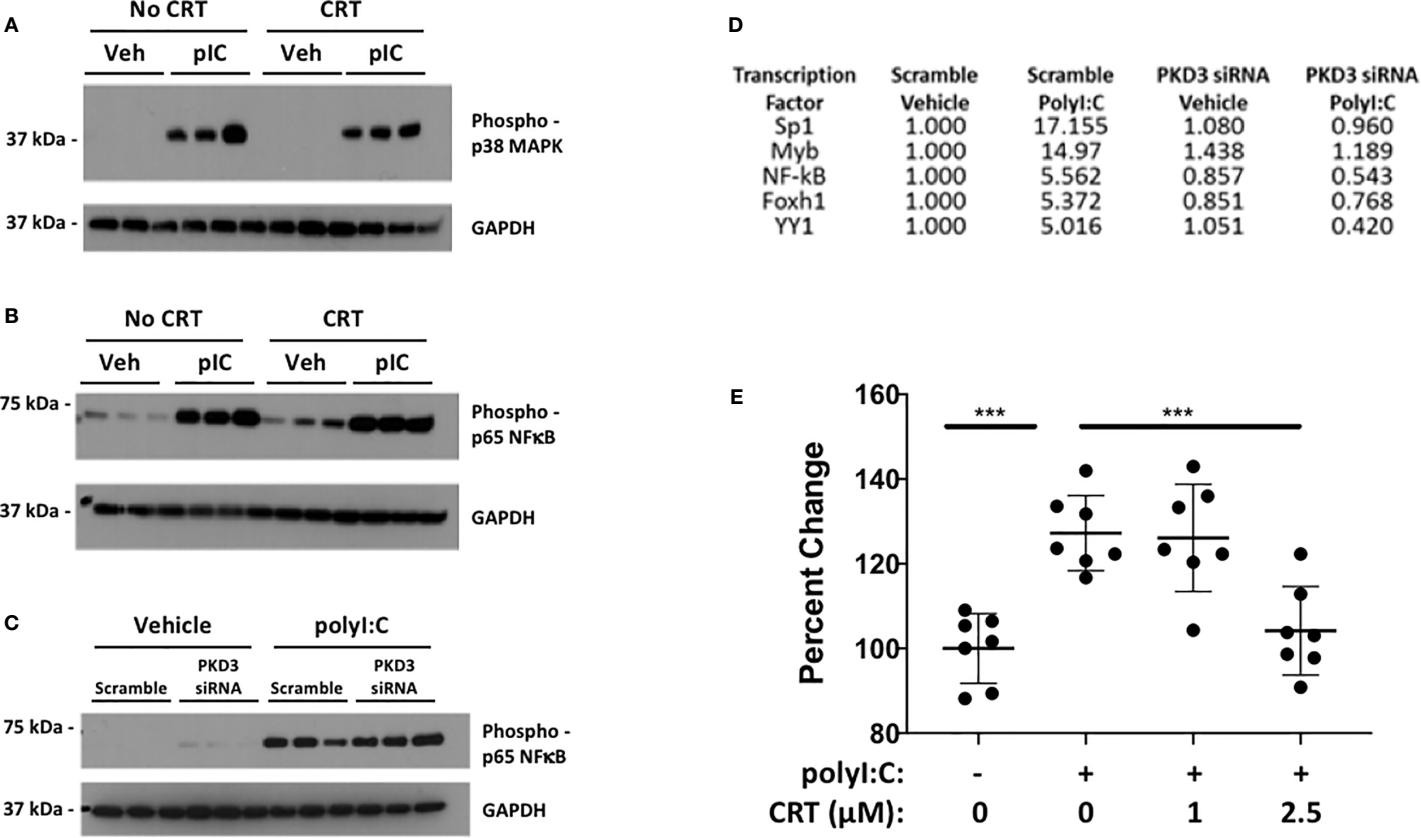
Protein kinase D promotes Sp1 activity over NF-kB or p38. 16HBE cells were pre-treated with 5 µM CRT for 2 h prior to stimulation with 5 µg/ml polyI:C as indicated for 20 mins. Whole cell lysates were analyzed for phospho-p65/NF-kB **(A)** and phospho-p38/MAPK **(B)** by Western blot. **(C)** 16HBE cells were transfected with siRNA as in **Figure 4** prior stimulation with 5 µg/ml polyI:C for 15 mins and whole cell extracts analyzed by Western. Three independent wells treated and analyzed for each condition (replicates denoted by “1, 2, 3”). **(D)** PKD3 was knocked down in 16HBE cells with 20 pmol siRNA overnight. Cells were then treated with 5 μg/ml polyI:C for 15min and nuclear extracts analyzed for activation of a panel of transcription factors using an unbiased DNA-binding assay kit (TF Profiling Plate Array 1 Kit, Signosis, see Methods). The top 5 transcription factors induced by polyI:C and inhibited by PKD3 siRNA knock-down are shown. See **Supplemental Table 1** for complete list. **(E)** 16HBE cells were pretreated 2h with 5 μM CRT before stimulation with polyI:C (5 μg/ml) for 5 min. Nuclear extract was harvested and Sp1 activity analyzed *via* DNA binding assay (RayBiotech). Results of two independent experiments are shown. *** indicates p<0.001 determined using one way ANOVA followed by Tukey’s t-test.

Several reports link PKD to activation of NF-kB and/or p38/MAPK in inflammatory contexts. We therefore tested the ability of PKD to phosphorylate NF-kB p65 and p38/MAPK in polyI:C-stimulated 16HBE cells (**Figure 7**). While polyI:C effectively promoted phosphorylation of NF-kB p65 and p38/MAPK, this was not blocked by CRT treatment (**Figures 7A, B**). Similarly, siRNA-mediated knock-down of PKD3 (see **Figure 4**) did not inhibit polyI:C-induced NF-kB p65 phosphorylation (**Figure 7C**). This led us to broaden our search for transcription factors activated by polyI:C, which were also inhibited by PKD3-knockdown. We performed an unbiased transcription factor array assay using nuclear extracts from polyI:C-treated 16HBE cells transfected with either scramble (control) or PKD3 siRNA constructs (see Methods). Out of 48 factors examined, 18 were induced at least two-fold by polyI:C (Sp1, Myb, NF-kB, FAST-1, YY1, GAS/ISRE, CAR, TCF/LEF, PXR, ER, TR, AP1, STAT6, SMAD, C/EBP, STAT3, NF-1 and HNF4) (**Supplemental Table 1**). The top five (**Figure 7D**), were highly activated by polyI:C treatment, and polyI:C-induced activation each of the factors shown was markedly attenuated by PKD3 siRNA knock-down (**Figure 7D**). Comparing **Figures 7C, D** suggests that PKD3 is involved in NF-kB activation independently of p65 phosphorylation, and that Sp1 may be a target of PKD activity following dsRNA challenge. To further determine if Sp1 is activated by polyI:C in a PKD-dependent manner, we performed a Sp1 DNA-binding assay with nuclear extracts from 16HBE cells treated with CRT +/− polyI:C. Sp1 DNA-binding was significantly enhanced by polyI:C stimulation, and this was effectively limited by CRT (**Figure 7E**). Finally, we performed Sp1 siRNA knock-down to determine whether Sp1 contributed to cytokine production following polyI:C challenge. As shown in **Supplemental Figure 6**, partial knockdown of Sp1 attenuated the production of IFN-lambda after polyI:C exposure. Taken together, these data indicate that polyI:C activates PKD which promotes cytokine production at least in part *via* Sp1 activation.

### PKD Is a Potential Target for New Antiviral Treatments

**Figure 8:**
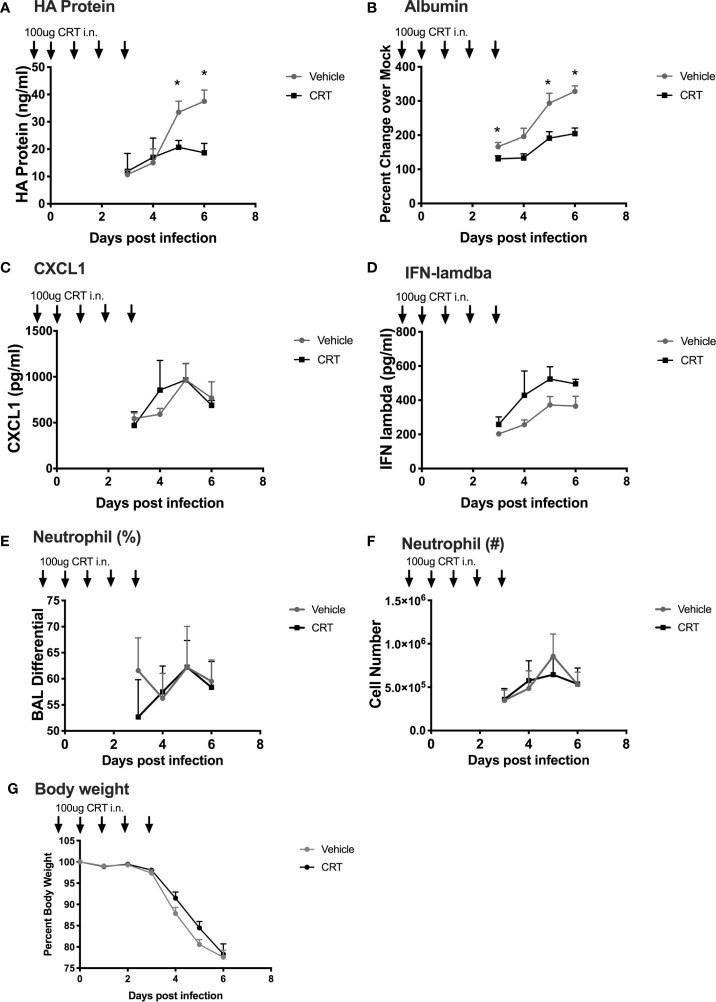
Inhibiting PKD restricts Influenza A Viral production and promotes barrier integrity in mice. C57BL/6 mice were dosed with CRT 1 day prior, day of, and 3 days after infection with 200 PFU IAV (PR/8). 5 mice per group were sacrificed days 3-6 post infection and BALF analyzed for **(A)** viral HA protein level (ELISA), **(B)** albumin (ELISA), **(C, D)** CXCL1 and IFN-lambda levels, **(E, F)** neutrophil percentages and total number, and **(G)** weight loss. Data are mean ± standard deviation. Each point represents the average of 5 mice per group per day (N=20 day 3, 15 day 4, 10 day 5, 5 day 6). One way ANOVA followed by Student’s t-test. *p < 0.05.

Our experiments point to a novel role for PKD in regulating not only epithelial barrier disruption, but also neutrophil influx into the lung after polyI:C challenge. These data suggest that inhibiting PKD might ameliorate airway inflammation during respiratory viral infections. However, we also noted that PKD inhibition lowered IFN-lambda production, a key cytokine involved in anti-viral defenses (19, 20). Therefore, the effect of blocking PKD in the context of respiratory viral infections *in vivo* was difficult to predict. In order to determine whether inhibiting PKD impacted the outcome of a viral infection, we used a mouse model of influenza A virus (IAV) infection. IAV is a substantial cause of morbidity and mortality worldwide, and directly infects airway epithelial cells. To investigate the role of PKD in IAV infection, we administered CRT intranasally to C57BL/6 mice one day prior and through day 3 post infection with a lethal dose of IAV (200 PFU of PR/8). There was no significant effect of CRT on IAV-induced mortality (data not shown). CRT treatment attenuated peak IAV levels as determined by ELISA for viral HA protein in BAL fluid (**Figure 8A**). CRT also promoted barrier integrity as CRT-treated mice had less albumin in BAL fluid (**Figure 8B**), consistent with data from polyI:C-treated mice. In contrast, CRT did not inhibit the production of CXCL1 or IFN-lambda in IAV-infected mice (**Figures 8C, D**), alter leukocyte composition in BAL fluid (**Figures 8E, F**), or affect IAV-induced weight loss (**Figure 8G**). These data suggest that inhibiting PKD may limit viral replication during IAV infection in addition to its role in barrier function. To determine whether administering CRT could have a therapeutic effect on IAV infection, we repeated experiments in separate groups of mice, and administered CRT i.n. the day of or one day after IAV infection. Using this therapeutic dosing model, CRT did not significantly lowered viral protein levels, neutrophil influx, or BAL albumin levels (**Supplementary Figure 7**).

**Figure 9:**
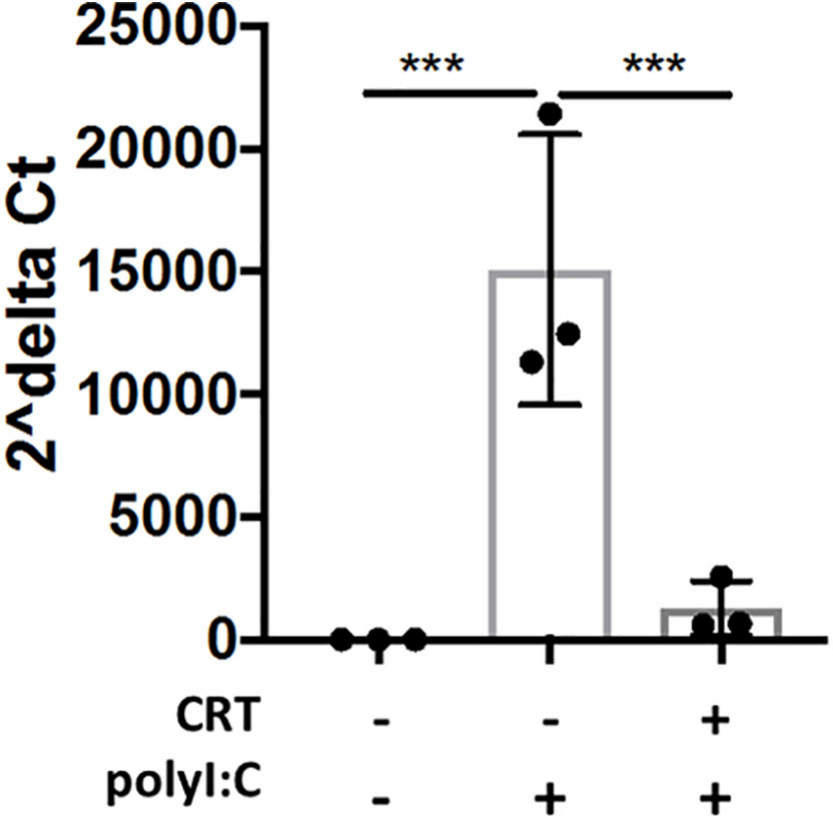
Inhibiting PKD restricts Influenza A Viral production in cell culture. 16HBE cells were pretreated 2h with 1 μM CRT or vehicle and infected 1hr with Influenza A (PR/8) at MOI 0.2. CRT was maintained in post-infection media. 48h post-infection, cell lysate and supernatant was collected and RNA was isolated using E.Z.N.A. kit (Omega). Levels of viral M protein mRNA were analyzed with RT-PCR. Data are mean ± standard deviation. Each point represents an individually treated well from 1–3 independent experiments. One way ANOVA followed by unpaired Tukey’s multiple comparisons test. ***p < 0.001.

In order to determine whether inhibiting PKD attenuated viral replication in airway epithelial cells, we next studied IAV replication *in vitro*. We infected 16HBE cells with low MOI (0.2) of IAV, and studied viral replication by RT-PCR using primers detecting IAV M protein mRNA levels. Interestingly, CRT treatment significantly limited viral mRNA levels (**Figure 9**), suggesting CRT may have direct anti-viral activity against IAV.

## Discussion

We and others have previously reported a role for protein kinase D (PKD) in regulating the barrier function of airway epithelial cell monolayers *in vitro* (3–5). Here we investigated if PKD regulates epithelial barrier function *in vivo*, and studied the role of PKD in additional aspects of airway epithelial cell response to acute airway inflammation. We found that PKD inhibition prevented polyI:C-induced barrier disruption *in vivo*, and observed a dose-dependent reduction of antiviral and pro-inflammatory cytokines/chemokines in both cell culture and mouse models of viral inflammation. Mechanistically, we demonstrated that PKD activity promotes cytokine mRNA transcription at least in part *via* Sp1 activation. Using CC10-Cre driven deletion, we report that conditional deletion of PKD3 in the airway partly attenuated dsRNA-induced barrier dysfunction, implicating an epithelial-intrinsic role for PKD3 *in vivo*. Finally, we show that targeting PKD promoted barrier integrity and attenuated viral protein production during lethal IAV infection, pointing to PKD as a possible novel target in the fight against influenza.

Regulation of airway epithelial barrier integrity is emerging as an important checkpoint in respiratory viral infections and lung inflammation, and our results establish a novel role for PKD in this process *in vivo.* Previous studies have implicated PKD3 in mediating barrier disruption in human airway epithelial cell lines. For example, siRNA-mediated knock down of PKD3 in 16HBE cells promotes barrier integrity by promoting maintenance of claudin-1 protein levels (4). Alternatively, PKD can phosphorylate actin-remodeling proteins (21), which can destabilize apical junctional complexes by promoting tight junction endocytosis. There is precedence for the idea that PKD can promote barrier dysfunction *via* actin cytoskeletal remodeling in models of acute lung injury (22). Future studies are needed to investigate the precise mechanism(s) by which PKD3 regulates airway barrier structures *in vivo* during viral infections.

PKD inhibition strikingly attenuated pro-inflammatory cytokine levels after dsRNA challenge (**Figure 2**). The role of PKD in mediating pro-inflammatory signaling has become increasingly apparent over the last decade, with several investigators linking PKD activity to activation of NF-kB and/or p38/MAPK activity (13, 23–26). However, inhibition or knock-down of PKD3 did not attenuate phosphorylation of NF-kB p65 or p38/MAPK in polyI:C-treated 16HBE cells (**Figure 7**). Instead, an unbiased transcription factor assay identified Sp1 as a potential transcription factor target of PKD activity, and a subsequent Sp1 binding assay confirmed that polyI:C stimulates Sp1 activity in a PKD-dependent manner. Sp1 is known to be activated in response to viral infections (27, 28), and PKD-dependent Sp1 activation was previously observed in human embryonic kidney cells infected with Kaposi Sarcoma Virus (29). Future studies investigating the role of PKD in Sp1-mediatd anti-viral innate immune responses should be insightful.

Although PKD has been previously implicated in cell activation downstream of Myd88-dependent pattern recognition receptors (11, 13, 26, 30), PKD activity has not previously been linked to dsRNA-dependent immune signaling pathways. In one prior study, polyI:C was unable to stimulate PKD activation in macrophages (13); however, we have observed a highly reproducible induction of PKD activity following polyI:C challenge in airway epithelial cells. This apparent contrast underscores the cell-type and isoform specific nature of PKD signaling (9). Although epithelial-specific PKD3 deletion significantly attenuated barrier dysfunction in the lung following polyI:C challenge, we have not ruled out a possible contribution of other PKD isoforms in airway epithelial cytokine production or subsequent leukocyte recruitment. Similarly, the partial protection from barrier disruption in the CC10-Cre x PKD3-floxed mice compared to CRT-treated mice suggests that other PKD isoform(s) and/or cell types contribute to regulation barrier integrity *in vivo*. These potential isoform-specific differences become especially intriguing in light of our recent finding that acute challenge with polyI:C promotes barrier disruption *via* TLR3, and cytokine production/airway inflammation *via* MDA5 signaling (15). It is therefore possible that PKD3 may mediate barrier disruption downstream of TLR3, while PKD1/2 may contribute to cytokine production downstream MDA5. If so, then isoform-specific PKD inhibitors could be used to specifically modulate either barrier function or inflammatory cell recruitment.

Our data add to the growing evidence that targeting PKD may restrict spread of several viruses. For example, inhibiting PKD can suppress the replication of rhinovirus (31), Kaposi sarcoma-associated herpesvirus (29), and Herpes simplex virus type 1 (32, 33) *in vitro*. The mechanisms by which inhibiting PKD restricts viral infection are not entirely clear. Using human 16HBE epithelial cells, we found that PKD inhibition (or PKD3 siRNA knock-down) human 16HBE cells blocked the production of IFN-lambda, a cytokine critical for early innate immune responses following IAV infection (19, 20, 34). It is possible that PKD acts on host antiviral proteins such as TRIM29 to regulate IFN levels (35). However, CRT did not significantly lower IFN levels in IAV-infected mice, and we recovered less IAV HA protein in BAL fluids from CRT-treated mice. As neither levels of viral protein nor mRNA can distinguish between productive and non-productive infection, future work using viral titers will be needed to precisely define how CRT restricts viral infection. However, the fact that CRT-treatment limited IAV infection in human airway epithelial cell line 16HBE, indicates that PKD is likely directly involved in the IAV lifecycle. CRT was more effective when administered before influenza infection in our model, compared with therapeutic dosing started after infection. In future experiments, it will be important to vary the dose, route and timing of CRT administration relative to influenza infection in order to determine the therapeutic potential of this approach.

Although CRT inhibited neutrophil influx into the lung in response to inhaled polyI:C, neutrophil numbers in influenza-infected mice were not significantly impacted by CRT. Similarly, whereas BAL CXCL1 and IFN-lambda levels were attenuated bv CRT in polyI:C challenged mice, the levels of these cytokines were not inhibited by CRT following influenza infection. These differences are likely due to the fact live replicating IAV engages multiple innate immune pathways and cell types (36) beyond those activated by inhaled dsRNA. Furthermore, inhaled polyI:C also does not induce an antigen-specific adaptive response. These observations underscore the importance of comparing live virus with any synthetic activator of a viral sensing pathway.

Taken together, our data establish a novel role for PKD3 in regulation of airway epithelial responses to viral infections, and indicate that targeting PKD has potential as a novel antiviral prophylactic strategy.

## Data Availability Statement

The raw data supporting the conclusions of this article will be made available by the authors, without undue reservation.

## Ethics Statement

The animal study was reviewed and approved by The Institutional Animal Care and Use Committee at the University of Rochester.

## Author Contributions

SG conceived of the study. Experimental design was led by SG, JV, and TC, though all authors contributed to experimental design and statistical analysis. JV conducted the experiments and was aided in data collection and data analysis by SH, SE, KS, CM, and ET. DT provided guidance about experiments with influenza viruses and supplied the virus strain used. All authors contributed to the article and approved the submitted version.

## Funding

The project described was supported by Award Number R01 HL12424 from NIH/NHLBI, F31 HL14079501 from NIH/NHLBI, T32AI007285 from NIH/NIAID, T32 HL066988 from NIH/NHLBI, T32 ES007026 from NIH/NIEHS. The funders had no role in study design, data collection and analysis, decision to publish or preparation of the manuscript.

## Conflict of Interest

The authors declare that the research was conducted in the absence of any commercial or financial relationships that could be construed as a potential conflict of interest.
