# Supplementary material for: Inhibiting Protein Kinase D Promotes Airway Epithelial Barrier Integrity in Mouse Models of Influenza A Virus Infection
Source: Front Immunol. 2020 Dec 14;11:580401. doi: 10.3389/fimmu.2020.580401 (PMC7767883; doi:10.3389/fimmu.2020.580401)
Supplement: Supplementary file 1 [file DataSheet_1.pdf]

Supplementary Information
Frontiers in Immunology
Manuscript ID: 580401

Inhibiting protein kinase D promotes airway epithelial barrier integrity in mouse models of influenza A virus infection

Janelle M. Veazey[1], Sophia I. Eliseeva[2], Sara E. Hillman[2], Kristie Stiles[2], Timothy R. Smyth[3], Charlotte E. Morrissey[4], Erika J.Tillotson[5], Dave J. Topham[1], Timothy J. Chapman[6], Steve N. Georas[2*]

[1]Department of Microbiology and Immunology, University of Rochester, Rochester, NY, United States.
[2]Department of Medicine, Pulmonary and Critical Care, University of Rochester, Rochester, NY, United States
[3]Department of Environmental Medicine, University of Rochester, Rochester, NY, United States
[4]Department Biochemistry, Claremont McKenna College, Claremont, CA, United States
[5]Department of Biology, Cornell University, Ithaca, NY, United States
[6]Center for Infectious Disease and Immunology, Rochester Regional Health, Rochester, NY, United States

Correspondence:
Steve N. Georas, M.D.
Steve_georas@urmc.rochester.edu

Supplemental Figure 1

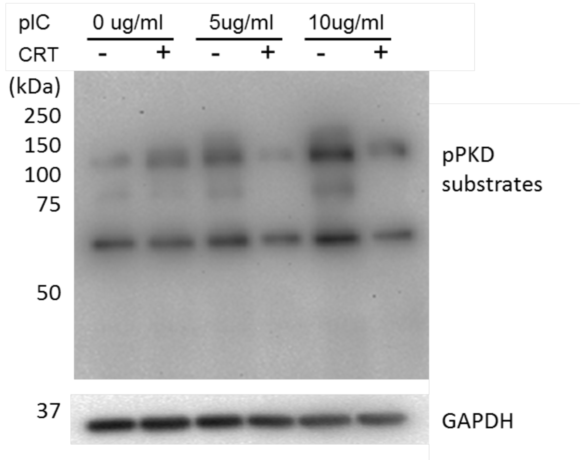

Supplemental Figure 1: polyI:C activates PKD and CRT inhibits PKD activation. 16HBE cells were pretreated for 2 hrs with 5 μM CRT, followed by stimulation with the indicated concentrations of polyI:C. After 24 hrs, cells were lysed in RIPA buffer and whole cell protein was analyzed with an antibody recognizing the phosphorylated region of PKD substrate (pMOTIF).

Supplemental Figure 2

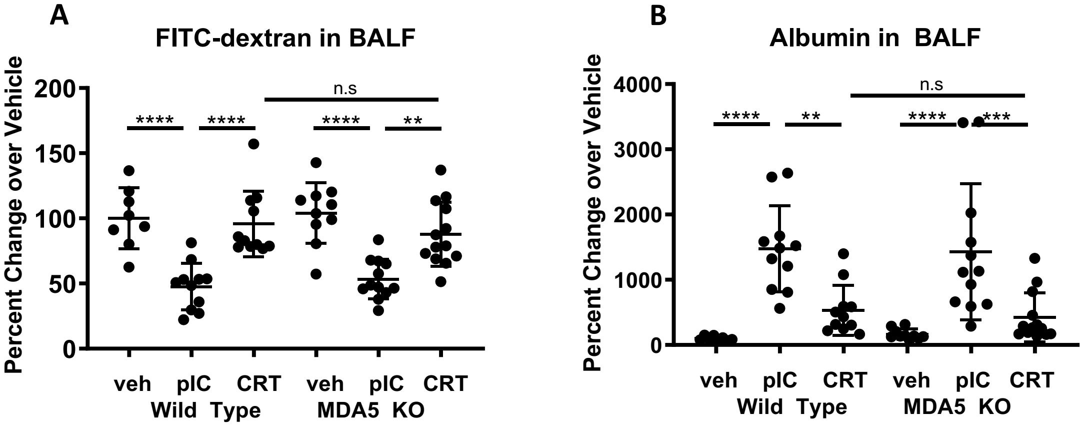

Supplemental Figure 2: CRT promotes barrier integrity in MDA5 knock-out mice. Wild type C57BL/6 mice or MDA5-/- mice were administered normal saline vehicle (Veh) or inhaled polyI:C (pIC) for three days. Twenty-four hours later, mice were sacrificed and bronchoalveolar lavage fluids (BALF) were collected. Barrier integrity was assessed via (A) loss of 4kDa FITC-dextran (outside/in leak), and (B) accumulation of albumin into the airspace (inside/out leak) as described in Methods. We previously reported that plyI:C-induced neutrophil influx was significantly attenuated in MDA5-/- mice, and not significantly different from saline-challenged control mice (Veazey et al., 2019). Data are mean ± SD pooled from two independent experiments using both male and female mice. Statistical significance was determined by two-way ANOVA with Tukey's multiple comparisons post-test analysis. $^{**}p<0.01$, $^{***}p<0.001$, $^{****}p<0.001$).

Supplemental Figure 3

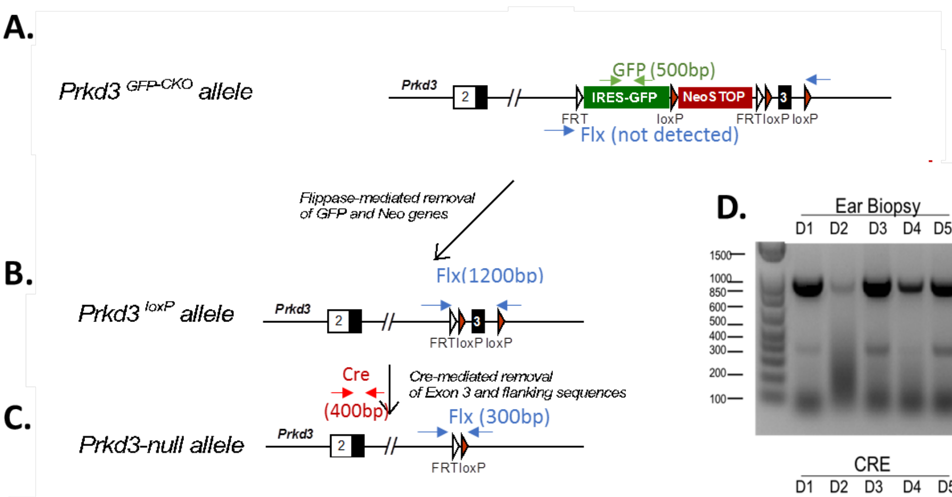

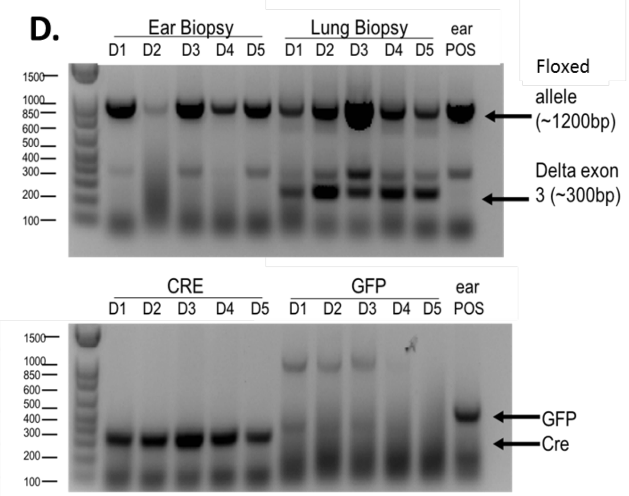

Primer	Sequence	Product Size
Flx-Forward	GATGAGTGGAACCGAATTCGAAG	1200bp (300bp after exon 3 deletion)
Flx-Reverse	CCAGCACTGCCACGTTCTCT	
Cre- Forward	CGGTCGATGCCACGAGTGATG	300bp
Cre-Reverse	ACGAACCTGGTCGAAATCAGTGCG	
GFP-Forward	GGCATCGTGCCCATCCTGATCGAGCTGA	500bp
GFP-Reverse	GCCAGCTGCACGCTGCCATCCTCGATGT	

PCR protocols:

Floxed allele
94°C, 5min
94°C, 30sec
52°C, 40sec
72°C, 1min 45sec
x36
72°C, 5min

Cre and GFP
94°C, 5min
94°C, 30sec
52°C, 25sec
72°C, 40sec
x40
72°C, 5min

Supplemental Figure 3: Genotyping strategy for CC10-Cre x PKD3-floxed mice. **(A-C)** Prkd3$^{GFP-CKO}$ mice were bread with Flippase mice to generate Prkd3^{loxP} (floxed) mice **(B)**. PKD3 floxed mice were bred with CC10-Cre mice to induce lung-specific conditional deletion **(C)**. **(D)** DNA from ear punch biopsies was extracted (AccuStart II, Cat# 95135, Quanta Biosciences) and analyzed for the presence of the PKD floxed allele (band at 1200bp with Flx primer set, and lack of GFP), and for the presence of Cre. Lung biopsies were analyzed in experimental mice to confirm the successful deletion of PKD3 (band at 300bp with Flx primer set).

Supplemental Figure 4

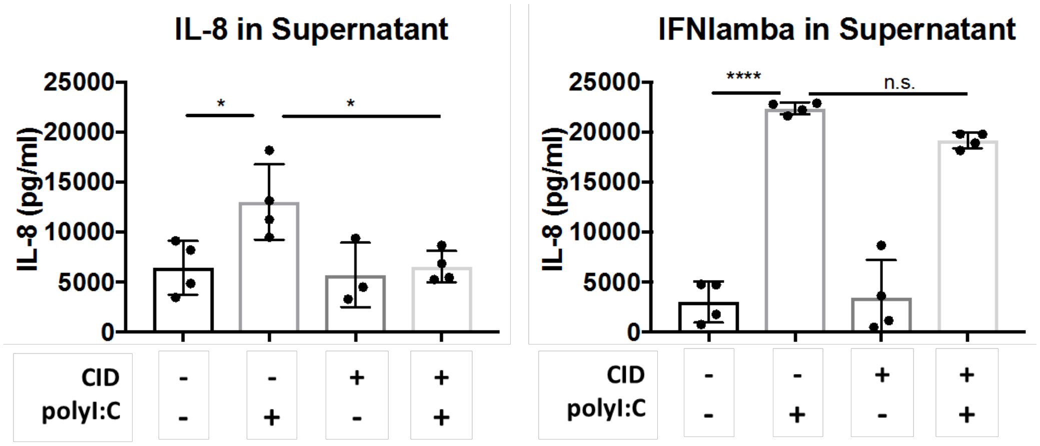

Supplemental Figure 4: CID limits IL-8 secretion. 16HBE cells were pretreated 2 hrs with 50 µM CID before polyI:C was spiked in to a final concentration 5 µg/ml. Supernatant and whole cell lysate was analyzed for IL-8 or IFNlambda via ELISA (R&D Bioscience). Data are mean ± standard deviation. Each point represents an individually treated well from 1 independent experiment. One way ANOVA followed by Tukey's t-test. *p<0.05, ****p<0.0001.

Supplemental Figure 5

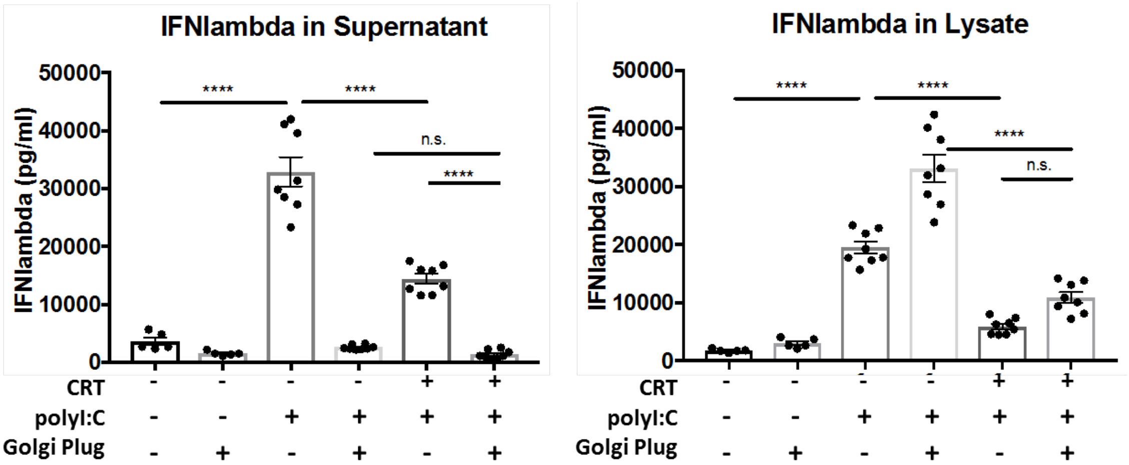

Supplemental Figure 5: Inhibiting PKD limits IFNlambda production. 16HBE cells were treated with Golgi Plug 30 min. prior to addition of CRT to a final concentration of 5 µM. 2 hrs later polyI:C was added to a final concentration of 5 µg/ml. After 6 hrs, supernatant and whole cell lysate was analyzed for IFNlambda via ELISA (R&D Bioscience). Data are mean ± standard deviation. Each point represents an individually treated well from 2 independent experiments. One way ANOVA followed by Tukey's t-test. *p<0.05, **p<0.01, ***p<0.001, ****p<0.0001

Supplemental Figure 6

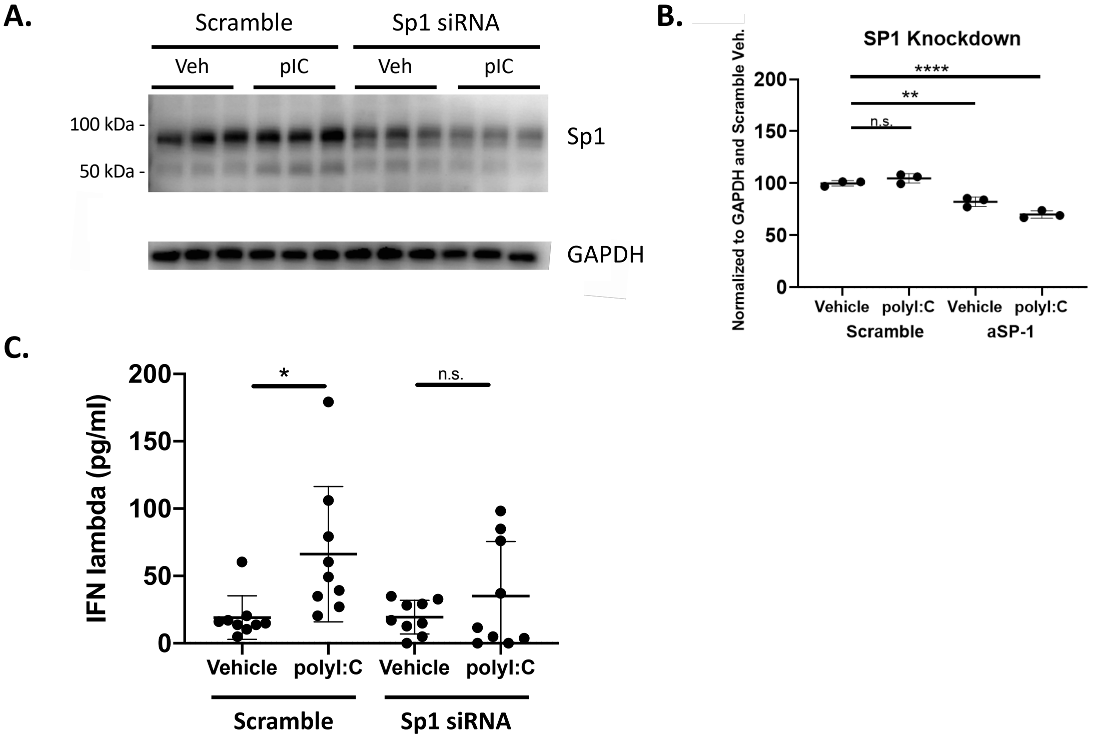

Supplemental Figure 6: siRNA-mediated knock down of Sp1 partially reduces Sp1 levels and polyI:C-induced interferon-lamda production. 16HBE cells were transfected with 20 pmol siRNA targeting Sp1 prior to 6hr stimulation with 5 µg/ml polyI:C. **(A)** siRNA-mediated knock down of Sp1 in 16HBE cells was confirmed via Western blot. **(B)** Percent Sp1 knockdown was determined by quantifying band intensity with Image J (normalized to GAPDH loading control and to scramble vehicle-treated). Data representative of three independent trials. (C) IFN-l production was analyzed by ELISA. Results are mean±SD of three independent experiments, analyzed by one way ANOVA followed by Tukey's t-test., $^{**}p<0.01$, $^{***}p<0.001$, $^{****}p<0.0001$.

Supplemental Figure 7

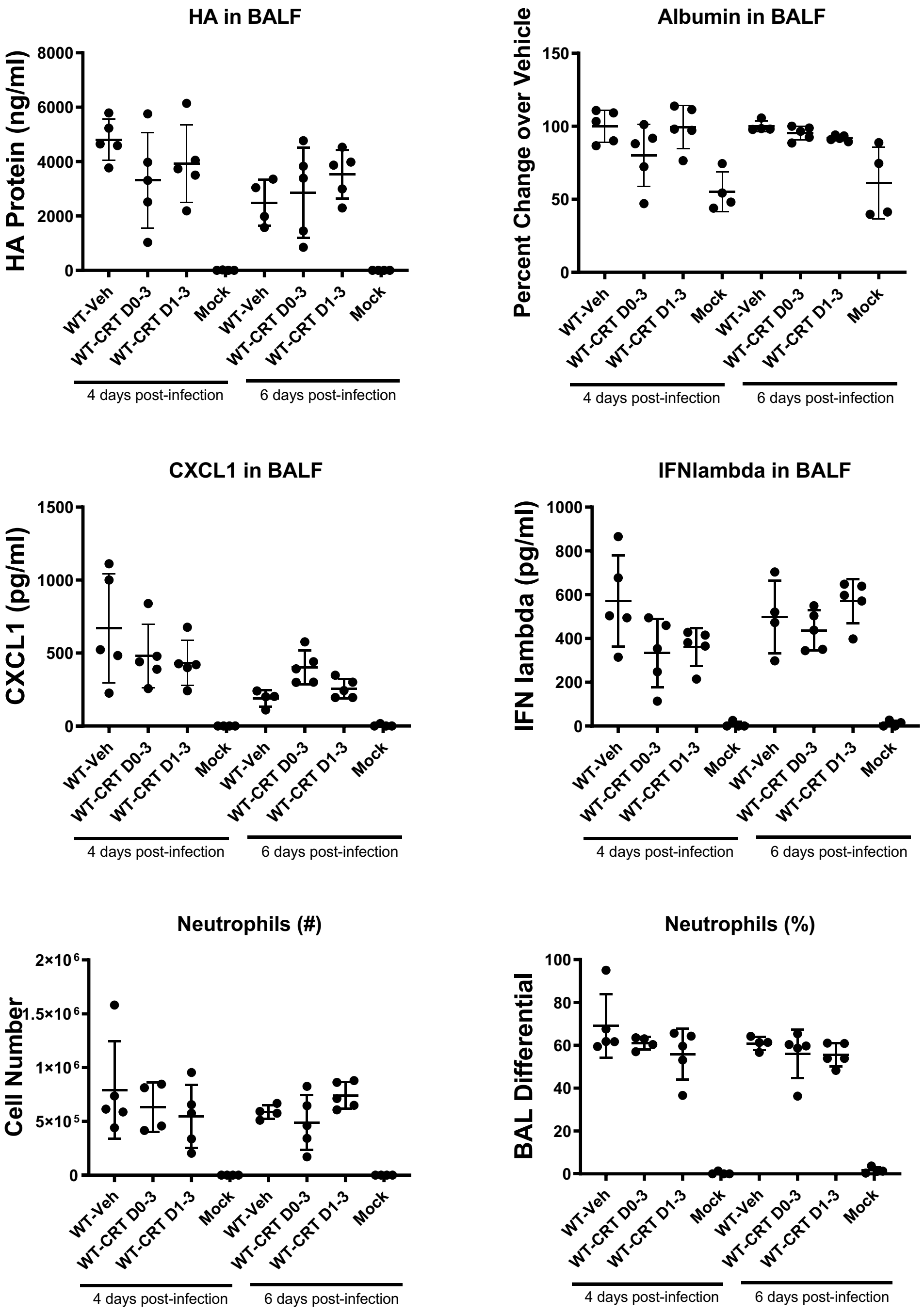

Supplemental Figure 7: Inhibiting PKD at the time of, or 1 day post infection, does not significantly limit Influenza A Viral production or barrier disruption in mice. C57BL/6 mice were dosed with CRT day of, and 1-3 days after infection with 200 PFU IAV (PR/8). 5 mice per group were sacrificed days 4 and day 6 post infection. BALF was analyzed for **(A)** viral HA protein level (ELISA), **(B)** albumin (ELISA), **(C-D)** IL-8 and IFN-lambda (ELISA), and **(E-F)** neutrophils (cytospin). Data are mean ± standard deviation. Each point represents the average of 5 mice per group per day of one independent experiment. One way ANOVA followed by Tukey's t-test.

Supplemental Table 1

Transcription Factor	Scramble Vehicle	Scramble PolyI:C	PKD3 siRNA Vehicle	PKD3 siRNA PolyI:C	Transcription Factor	Scramble Vehicle	Scramble PolyI:C	PKD3 siRNA Vehicle	PKD3 siRNA PolyI:C
TFIID	1.000	**70.173***	3.468	4.395	HIF	1.000	1.693	1.218	0.614
Sp1	1.000	**17.155***	1.080	0.960	ATF2	1.000	1.548	2.615	0.658
Myb	1.000	**14.972***	1.438	1.189	PAX-5	1.000	1.496	1.375	1.050
NFkB	1.000	**5.562***	0.857	0.543	STAT1	1.000	1.446	3.107	2.002
FAST-1	1.000	**5.372***	0.851	0.768	Ets	1.000	1.442	3.544	1.732
YY1	1.000	**5.016***	1.051	0.420	E2F-1	1.000	1.427	2.078	1.025
GAS/ISRE	1.000	**2.984***	17.173	12.897	SATB1	1.000	1.376	3.808	3.107
CAR	1.000	**2.850***	2.004	1.006	NF-E2	1.000	1.329	2.673	2.397
TCF/LEF	1.000	**2.713***	4.818	2.697	Myc-Maz	1.000	1.248	1.615	1.145
PXR	1.000	**2.643***	3.970	1.748	EGR	1.000	1.185	2.681	1.185
ER	1.000	**2.497***	3.598	2.168	NFAT	1.000	1.150	5.903	2.053
TR	1.000	**2.423***	4.320	1.284	Oct 4	1.000	1.149	0.389	0.809
AP1	1.000	**2.418***	0.806	0.956	AP2	1.000	1.121	0.697	1.127
STAT6	1.000	**2.309***	1.091	0.440	PPAR	1.000	1.094	1.008	2.314
SMAD	1.000	**2.194***	1.074	0.574	IRF	1.000	1.089	1.422	0.800
C\EBP	1.000	**2.152***	3.922	2.956	CREB	1.000	0.983	3.218	1.569
STAT3	1.000	**2.051***	1.797	13.253	GATA	1.000	0.970	0.828	0.702
NF-1	1.000	**2.032***	2.184	1.067	p53	1.000	0.839	0.722	1.433
HNF4	1.000	**2.019***	2.377	1.377	Pit	1.000	0.692	1.318	0.836
STAT5	1.000	1.987	0.794	0.645	CDP	1.000	0.678	0.770	0.540
Brn-3	1.000	1.939	0.602	1.276	MEF2	1.000	0.676	0.777	0.593
SRE	1.000	1.863	3.150	2.157	AR	1.000	0.641	0.539	0.396
Pbx1	1.000	1.748	7.523	8.836	GR/PR	1.000	0.503	0.645	1.632
CBF	1.000	1.702	1.250	0.922	STAT4	1.000	**0.189***	0.188	1.467

Supplemental Table 1: Transcription factors activated by polyI:C. PKD3 was knocked down in 16HBE cells with 20 pmol siRNA overnight. Cells were then treated with 5 µg/ml polyI:C for 15min and nuclear extracts run according to TF Activation Profiling Plate Array 1 Kit (Signosis). Results from one experiment. **(C)**16HBE cells were pretreated 2hrs with 5 µM CRT before polyI:C was spiked in to a final concentration 5 µg/ml. 5 min. later nuclear extract was harvested and Sp1 activity analyzed via DNA binding assay (RayBiotech). Results pooled from two independent experiments. 2 fold increase or decrease from scramble-vehicle is $p<0.05$ (**bold***).
